# Patient-specific computational fluid dynamics analysis of portal vein hemodynamics before and after balloon angioplasty following living donor liver transplantation: a proof-of-concept study

**DOI:** 10.3389/fbioe.2026.1782867

**Published:** 2026-04-24

**Authors:** Fengleng Yang, Chihan Peng, Yan Luo, Hong Xiao, Lin Ma

**Affiliations:** 1 Department of Ultrasound, West China Hospital, Sichuan University, Chengdu, Sichuan, China; 2 State Key Laboratory of Hydraulics and Mountain River Engineering, Sichuan University, Chengdu, Sichuan, China; 3 Department of Ultrasound, West China Tian Fu Hospital, Sichuan university, Chengdu, Sichuan, China

**Keywords:** balloon angioplasty, computational fluid dynamics (CFD), hemodynamics, liver transplantation, portal vein stenosis (PVS)

## Abstract

Portal vein stenosis (PVS) is a clinically significant complication after living donor liver transplantation (LDLT) that can compromise portal hemodynamics and graft perfusion. Because conventional imaging primarily assesses morphology and local velocities, it provides limited insight into system-level hemodynamic alterations. Here, we developed a longitudinal, patient-specific computational fluid dynamics (CFD) framework to quantitatively evaluate portal venous hemodynamics before and after balloon angioplasty in an LDLT recipient with PVS. Patient-specific portal venous geometry was reconstructed from imaging data, with inlet conditions derived from Doppler ultrasound and outlet conditions modeled using a three-element Windkessel model. Key hemodynamic metrics—including velocity ratio, branch flow distribution, trans-stenotic pressure gradient, and wall shear stress—were analyzed before surgery and at 1 week and 6 months post-surgery. Balloon angioplasty markedly reduced velocity amplification and pressure gradients across the stenosis, normalized wall shear stress patterns, and induced progressive redistribution of intrahepatic portal flow, indicating system-level hemodynamic remodeling. This proof-of-concept study demonstrates that patient-specific CFD provides complementary functional information beyond conventional imaging and suggests its potential to facilitate longitudinal monitoring and early identification of hemodynamic changes, serving as a complementary tool for postoperative surveillance.

## 1 Introduction

Liver transplantation remains the only curative treatment for end-stage liver disease, and the increasing role of living donor liver transplantation (LDLT) has major global clinical implications. LDLT has substantially expanded the donor pool and reduced waitlist mortality, becoming the predominant transplant modality in many Asian countries and an increasingly important option in Europe and North America as deceased-donor shortages persist (Terrault et al., 2023; Kasahara and Sakamoto, 2025; Jackson et al., 2022). Optimizing the management strategies for recipient complications is essential to the success of LDLT.

Portal vein stenosis (PVS) is a clinically important vascular complication after LDLT that can compromise graft perfusion and long-term outcomes. PVS occurs with a reported incidence of approximately 8%–14% and most commonly results from donor–recipient portal vein (PV) diameter mismatch or intraoperative factors such as torsion, angulation, or anastomotic tension (Ueda et al., 2005; Darwish et al., 2006). When stenosis is severe, portal inflow reduction can precipitate graft dysfunction or failure, whereas milder lesions may be clinically silent; therefore, early detection and timely endovascular therapy (for example, balloon angioplasty) are critical to preserve graft function (Xiao et al., 2012; Narita et al., 2019; Ohgi et al., 2019; Kim et al., 2019). Because these clinical consequences depend on haemodynamic disturbance rather than anatomy alone, a shift from purely anatomical assessment toward hemodynamic evaluation is necessary to guide durable management.

Conventional vascular imaging modalities commonly used in clinical practice inadequately characterize the complex hemodynamics that governs PVS progression and post-intervention adaptation. Digital subtraction angiography is invasive, computed tomography angiography (CTA) provides mainly morphological information, and ultrasound offers limited hemodynamic metrics. Collectively, these modalities fail to capture complex three-dimensional flow patterns, energy loss, branch perfusion imbalance, and wall mechanical stimuli. Because vascular remodeling and long-term patency are governed by local hemodynamics—such as wall shear stress (WSS), pressure gradients, and flow oscillation—these biomechanical factors form the critical link between surgical intervention and biological response. This limitation constrains precise monitoring and personalized decision-making after PVS treatment.

Computational fluid dynamics (CFD) enables noninvasive, quantitative assessment of global hemodynamics, bridging imaging-derived anatomy and the local hemodynamics that drive biological responses (Riedel et al., 2024; Xie et al., 2024). However, most prior CFD studies are cross-sectional, limited to single pre- or postoperative time points, and therefore fail to capture the longitudinal hemodynamic evolution from stenosis relief to long-term adaptation. Because hemodynamic patterns may change between early and later postoperative phases, longitudinal patient-specific CFD incorporating physiological boundary conditions is essential to elucidate mechanisms of durable hemodynamic improvement or restenosis and to inform clinical management of PVS after LDLT.

This proof-of-concept study presents a longitudinal, patient-specific CFD framework to quantitatively assess the hemodynamic effects of balloon dilatation for PVS following LDLT. Unlike conventional imaging-based evaluations, our framework provides quantitative, clinically interpretable hemodynamic metrics that reliably track therapeutic efficacy and temporal evolution of vascular adaptation, holding promise for potential applications in monitoring vascular complications following LDLT.

## 2 Materials and methods

### 2.1 Clinical data

A 55-year-old male who underwent balloon angioplasty for PVS after LDLT in 2015 at West China Hospital of Sichuan University was included in this study. The patient had received right-lobe LDLT for liver failure, with end-to-end anastomosis performed between the donor and recipient PVs. On the 13th day after liver transplantation, grayscale ultrasound revealed significant stenosis at the PV anastomosis site, with a width of approximately 5 mm. Doppler ultrasound demonstrated a markedly increased blood flow velocity at the stenosis, with velocities of 51.26 cm/s at the stenosis and 13.45 cm/s in the pre-stenotic region. Subsequent CTA further confirmed the presence of PVS.

### 2.2 Therapeutic interventions and follow-up

The patient underwent percutaneous transluminal balloon angioplasty for PVS under fluoroscopic guidance, followed by postoperative anticoagulation. The procedure was successful, with stable hemodynamics and favorable follow-up. Intraoperatively, the trans-stenotic pressure gradient measured using a pressure measurement catheter was 1.8 mmHg pre-dilation and 0.2 mmHg post-dilation. Preoperatively, the stenotic segment measured 5 mm with pre-stenotic dilatation of 15 mm. One week after angioplasty, the stenosis expanded to 8 mm and the pre-stenotic diameter decreased to 12 mm. At 6 months, further normalization of the pre-stenotic region to 11 mm indicated progressive geometric recovery. Corresponding doppler ultrasound-measured peak velocities at the stenotic and pre-stenotic sites were 14.13 and 13.32 cm/s at 1 week, and 14.89 and 15.13 cm/s at 6 months.

### 2.3 Three-dimensional (3D) reconstruction

The patient underwent a high-resolution 256-slice spiral CT (Revolution CT, GE Healthcare, Milwaukee, United States) scan with the following imaging parameters: an in-plane pixel resolution of 0.82 × 0.82 mm, a slice thickness of 1 mm, a slice spacing of 0.5 mm, and a matrix size of 512 × 512. The high-resolution images enable accurate reconstruction of the vascular geometry model.

**FIGURE 1:**
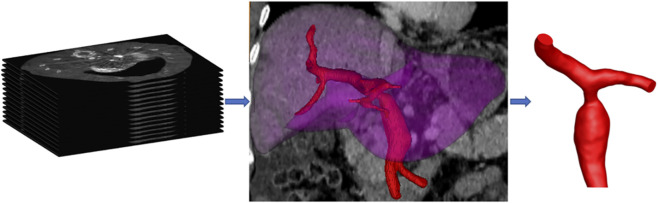
Schematic diagram of the three-dimensional geometric model reconstruction of the portal vein branch in the living donor liver transplant recipient with postoperative portal vein stenosis.

**FIGURE 2:**
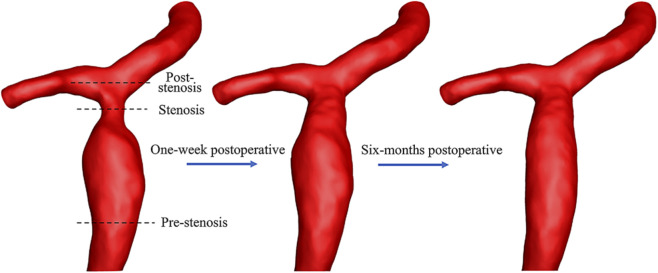
Three-dimensional models of the portal vein branch of the patient before and after balloon angioplasty.

Three-dimensional geometric models of the right PV branch were reconstructed using Mimics software (Materialise, Leuven, Belgium) for three time points: before surgery, 1 week post-surgery, and 6 months post-surgery. This software has been extensively validated for three-dimensional reconstruction of medical images (Xie et al., 2024; Wang et al., 2023), and in this study, the reconstructions were morphologically confirmed by a senior radiologist. The specific procedures were as follows: (1) The DICOM-formatted CTA were imported into Mimics software (Materialise, Leuven, Belgium). The main region of interest—the PV trunk and its primary branches—was segmented from the CT images based on pixel grayscale thresholds, followed by manual removal of extraneous tissues; (2) Subsequently, adjacent cross-sectional images were reconstructed and interpolated to generate spatially uniform cubic voxel parameters, thereby constructing the 3D model; (3) The 3D model was subsequently smoothed and refined to ensure surface continuity and anatomical fidelity, and was then rendered for stereoscopic visualization on the computer screen (Figures 1, 2). The preoperative stenotic segment measured 5.1 mm with a pre-stenotic dilatation of 15.2 mm. The downstream right anterior and posterior branches have maximum diameters of approximately 5.0 mm and 7.3 mm, respectively.

### 2.4 Meshing generation

**FIGURE 3:**
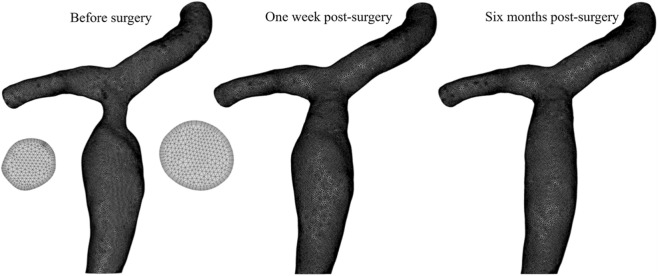
Mesh generation of the portal vein branch model before and after balloon angioplasty.

The 3D model was imported into ANSYS ICEM CFD (ANSYS Inc, Canonsburg, PA) for mesh generation. A hybrid mesh combining unstructured tetrahedral and hexahedral elements was employed, with local refinement applied to the boundary layer at the right PV wall. The first layer height was set at 0.065 mm, following an exponential growth pattern with a growth rate of 1.2 for a total of five layers (Figure 3). To ensure the accurate resolution of near-wall velocity gradients and WSS, the boundary layer mesh quality was evaluated. The maximum y + value was calculated to be approximately 1.66 (at the stenosis site). To ensure the computational results were independent of the mesh and to guarantee their accuracy, a grid independence verification was performed for all meshes. Using the maximum flow velocity in the PV, trans-stenotic pressure gradient, and maximum WSS at the stenosis as evaluation metric, the results showed that the when the maximum grid size was refined from 0.65 mm to 0.60 mm, the relative differences in all indicators were less than 1%, indicating that the solution is grid-independent. Table 1 presents the number of mesh elements generated with different mesh sizes and the corresponding hemodynamic parameters in the PV. Balancing computational accuracy with efficiency, the maximum mesh size for the right PVS model in this study was set to 0.65 mm.

**TABLE 1:** The number of mesh generated with different mesh sizes and the corresponding hemodynamic parameters in the portal vein.

Maximum grid size (mm)	Grid quantity	Maximum velocity in the portal vein (cm/s)	Trans-stenotic pressure gradient (Pa)	Maximum WSS at the stenosis (Pa)
0.85	752,596	47.55	188.42	27.93
0.75	786,410	47.97	193.85	29.15
0.65	859,995	48.91	197.13	30.21
0.60	927,816	48.84	195.22	30.49

### 2.5 Boundary condition coupling

**FIGURE 4:**
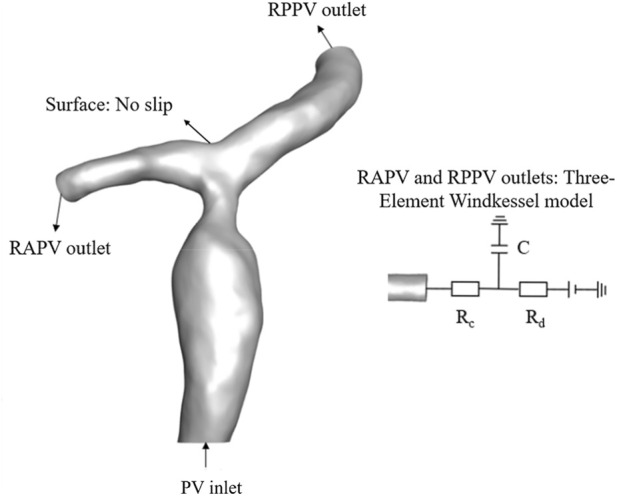
An illustration of the boundary conditions of the portal vein system in the computational fluid dynamics. simulations. PV: portal vein; RAPV, right anterior portal vein; RPPV, right posterior portal vein.

The selection of boundary conditions is critically important for the accuracy of numerical simulation results. Given that the pulsatility of portal venous flow is considered insignificant (George et al., 2015), we adopted a constant blood flow velocity for numerical simulations, following previous studies (Xie et al., 2024; Xiong et al., 2024). In clinical vascular assessment, peak velocity is a key metric. In this study, the inlet boundary condition was defined based on ultrasound-derived maximum velocity, enabling the calculation of the most extreme WSS and trans-stenotic pressure gradient (worst-case scenario); employing mean velocity would underestimate the hemodynamic impact of the stenosis. The outlet boundaries were coupled using a three-element Windkessel (WK3) model (Figure 4). The parameters of the WK3 model—namely, the characteristic impedance *R*_*c*_, peripheral resistance *R*_*d*_, and vascular compliance *C*—were determined based on clinical measurements and relevant published literature (Ho et al., 2012; Capoccia, 2015; S et al., 2024). The calculated parameter values for the WK3 model outlet boundaries are summarized in Table 2, with the downstream pressure *P*_*d*_ set to 0 Pa (a reference gauge pressure for the incompressible fluid solver to compute the relative pressure gradient, rather than an absolute physiological baseline). The vessel walls are modeled as rigid walls, and non-slip boundary conditions are applied at the vessel walls (Riedel et al., 2024; Xiong et al., 2024; Yin et al., 2022; S et al., 2024).

**TABLE 2:** Parameters of resistance and capacitance in the Three-Element Windkessel coupled model of the right portal vein.

Outlet	$R_{d}\left(\text{Pa}\cdot s/m^{3}\right)$	$R_{d}\left(\text{Pa}\cdot s/m^{3}\right)$	$C\left(m^{3}/\text{Pa}\right)$
RAPV	2.7175 × 10^8^	1.9017 × 10^9^	9.4916 × 10^−9^
RPPV	2.4875 × 10^8^	1.8697 × 10^9^	9.4916 × 10^−9^

RAPV; right anterior portal vein.

RPPV; right posterior portal vein.

### 2.6 Solver settings

Considering the low shear rate and pronounced non-Newtonian behavior in PVS, blood was modeled as an incompressible non-Newtonian fluid (ρ = 1,060 kg/m^3^). using the Carreau–Yasuda viscosity model. The maximum Reynolds number (Re = ρVD/μ) in this study was 740, which occurred at the preoperative stenosis. Since this value is well below 2000, the flow was considered to be laminar (S et al., 2024). The time step was set to 0.005 s for 1,000 steps, with convergence thresholds of 10^−5^ for all degrees of freedom. Flow rate and pressure at the first-order right PV branches were monitored throughout the simulation, and convergence was confirmed when the flow field reached a fully developed, quasi-steady state, and these parameters stabilized with minimal variation across successive time steps.

## 3 Results

### 3.1 PV flow field distribution

**FIGURE 5:**
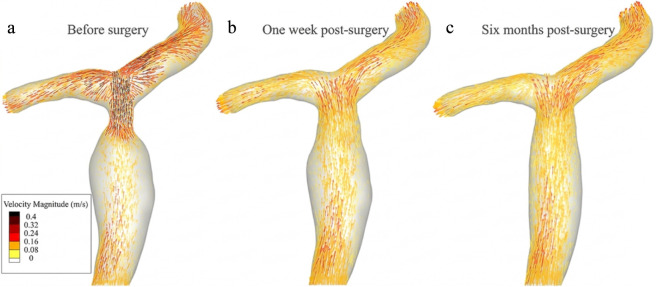
The portal vein blood flow velocity distribution of the patient before and after balloon angioplasty. **(a)** Before surgery, exhibiting a high-velocity jet; **(b)** One week post-surgery, showing acute flow restore; **(c)** Six months post-surgery, indicating long-term hemodynamic remodeling.

In the preoperative model, blood flow from the main PV passes through the stenotic segment into the branches, exhibiting three distinct hemodynamic patterns (Figure 5).

1. Pre-stenotic segment: Significant luminal dilation results in an expanded flow area, creating a buffer zone with markedly reduced flow velocity.
2. Stenotic segment: Abrupt luminal narrowing at the anastomosis leads to a significant reduction in the cross-sectional area. Consequently, blood flow velocity is significantly increased, forming a high-velocity jet in this region.
3. Post-stenotic segment: Immediately distal to the stenosis, the flow became disturbed, exhibiting laminar separation and recirculation at the bifurcation, leading to asymmetric velocity distribution in the right anterior and posterior PV branches—higher velocities along the inner walls and lower velocities along the outer walls.

**FIGURE 6:**
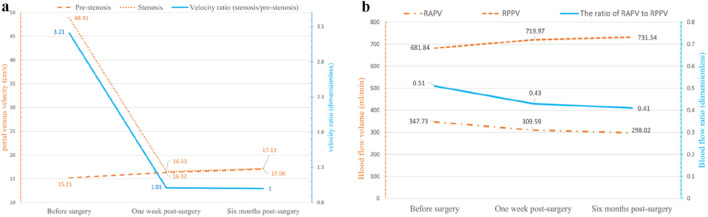
The portal vein flow velocity and blood flow in each branch before and after balloon angioplasty: **(a)** Blood flow velocity in different regions of the portal vein before and after balloon angioplasty. Following balloon angioplasty, the flow velocity at the stenotic segment of the portal vein was markedly reduced, with the velocity ratio between the stenotic and pre-stenotic regions approaching unity; **(b)** Blood flow in the right anterior and right posterior portal vein branches before and after balloon angioplasty. A progressive redistribution of blood flow between the RAPV and RPPV was observed in the postoperative period, indicating ongoing hemodynamic remodeling. RAPV: right anterior portal vein; RPPV: right posterior portal vein.

CFD simulation revealed that preoperative portal venous velocity (PVV) at the stenosis reached 48.91 cm/s (compared with 51.26 cm/s measured by ultrasound), while it was 15.21 cm/s in the pre-stenotic region. The resulting PVV ratio (stenotic site/upstream) was approximately 3.21 (Figure 6a), which is consistent with the results of Doppler ultrasound and validates the accuracy of this model. One week after surgery, the velocity at the original stenosis decreased to 16.53 cm/s, with a pre-stenotic velocity of 16.32 cm/s, resulting in a PVV ratio of about 1.01—a reduction of approximately 66.3% compared to the pre-surgery value. After 6 months post-surgery, the velocities at the stenotic and pre-stenotic sites were 17.13 cm/s and 17.06 cm/s, respectively, maintaining a ratio near 1.0. Although a slight increase in stenotic velocity was observed compared to the one-week measurement, overall hemodynamics showed further normalization.

Longitudinal comparison revealed two distinct phases of vascular recovery. Compared with the preoperative state, the high-velocity jet disappeared 1 week after surgery, accompanied by a rapid attenuation of downstream flow disturbances, highlighting the immediate mechanical effect of balloon angioplasty in relieving the stenosis. In contrast, between 1 week and 6 months post-surgery, the gradual normalization of the pre-stenotic dilated segment (from 12 mm to 11 mm) was accompanied by subtle adjustments in blood flow velocity (from 16.53 cm/s to 17.13 cm/s at the stenotic site). These medium-to long-term changes reflect an ongoing process of physiological vascular remodeling.

From the preoperative period to 1 week and further to 6 months post-surgery, the high-velocity jet flow at the stenotic segment gradually resolved. The pre-surgery blood flow in the right posterior branch of the PV was 681.84 mL/min, while that in the right anterior branch was 347.73 mL/min, yielding a flow ratio between the right anterior and posterior branches of approximately 0.51. At 1 week postoperatively, the blood flow in the right posterior branch measured 719.97 mL/min, and that in the right anterior branch was 309.59 mL/min, with a corresponding ratio of about 0.43. By 6 months after surgery, the blood flow in the right posterior branch reached 731.54 mL/min, and in the right anterior branch, it was 298.02 mL/min, resulting in a further decreased ratio of approximately 0.41 (Figure 6b).

### 3.2 PV WSS distribution

**FIGURE 7:**
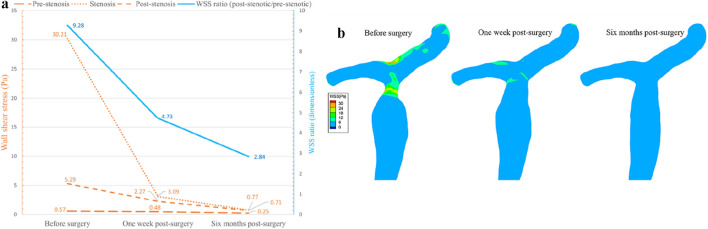
Changes in WSS within the portal vein branch before and after balloon angioplasty: **(a)** Following balloon angioplasty, WSS at the stenotic site within the portal vein exhibited a progressive decline, with the ratio of post-stenotic to pre-stenotic WSS continuously decreasing over time; **(b)** The portal vein wall shear stress distribution before and after balloon angioplasty. WSS: wall shear stress.

Before intervention, the maximum WSS at the stenosis reached 30.21 Pa, compared with 0.57 Pa in the pre-stenotic and 5.29 Pa in the post-stenotic regions (Figure 7a), yielding a WSS ratio (post-stenotic/pre-stenotic) of approximately 9.28. At 1 week postoperatively, the maximum WSS at the former stenotic site markedly decreased to 3.09 Pa, while the pre- and post-stenotic regions measured 0.48 Pa and 2.27 Pa, respectively. The corresponding WSS ratio declined to 4.73, representing reductions of approximately 89.8% in peak WSS and 49.0% in WSS ratio compared with preoperative levels. By 6 months after intervention, the maximum WSS at the stenosis further decreased to 0.77 Pa, with corresponding values of 0.25 Pa in the pre-stenotic and 0.71 Pa in the post-stenotic regions. The calculated WSS ratio was 2.84, reflecting an additional 75.1% reduction in peak WSS and a 40.2% decrease in WSS ratio relative to the one-week postoperative measurements. Although absolute WSS values changed modestly between 1 week and 6 months, the overall WSS distribution throughout the portal venous system exhibited progressive normalization, suggesting sustained hemodynamic stabilization following angioplasty (Figure 7b).

### 3.3 PV pressure distribution

**FIGURE 8:**
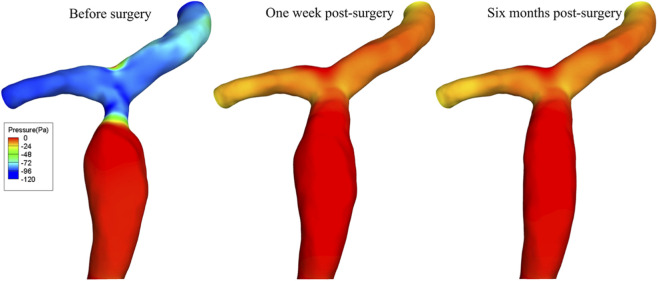
The portal vein pressure distribution of the patient before and after balloon angioplasty.

Before balloon angioplasty, a pronounced pressure gradient was present across the PVS. Following the surgery, effective dilation of the stenotic segment resulted in progressive hemodynamic restoration and a marked decline in the trans-stenotic pressure gradient (Figure 8). Based on the pressure measurement locations during balloon angioplasty, the cross-sectional area-averaged pressures at the pre- and post-stenotic regions were calculated respectively, and the difference between these two values represented the trans-stenotic pressure gradient. Because the downstream pressure in the Windkessel model was set to 0 Pa, the reported values represent relative pressure. CFD simulations demonstrated that the trans-stenotic pressure gradient was 197.13 Pa (≈1.48 mmHg) preoperatively, which was comparable to that measured via catheter (1.8 mmHg), thereby validating the accuracy of the model. At 1 week postoperatively, it decreased to 12.86 Pa, representing a 93.47% reduction. By 6 months after the procedure, the gradient further declined to 4.67 Pa, corresponding to an additional 63.69% decrease compared with the one-week value. The postoperative pressure distribution in the portal venous system has thus approached the physiological range observed in normal PV hemodynamics.

## 4 Discussion

This proof-of-concept study employed patient-specific CFD simulations to investigate the hemodynamic effects of percutaneous transluminal balloon dilatation on PVS following LDLT. By conducting CFD simulations longitudinally at multiple time points before and after the surgery, we demonstrated that the surgery resulted in marked improvements in portal venous hemodynamics and this improvement can be quantified and visualized. Importantly, these improvements were not confined to the stenotic segment but extended to downstream branches, and exhibited a temporal evolution from the early postoperative phase to mid-term period, complementing conventional imaging-based assessments. Tracking longitudinal hemodynamic trends may provide early warning signs before anatomical and functional changes occur in the PV, thereby assisting clinicians in optimizing postoperative management for these patients.

Preoperative CFD simulations demonstrated marked flow obstruction at the PVS, causing proximal blood pooling and elevated local wall pressure. Sustained circumferential stress may drive vascular remodeling and compensatory luminal dilation to reduce flow resistance. Abrupt luminal narrowing increased local flow velocity to preserve portal inflow, generating a high-velocity jet associated with elevated WSS and jet impingement, which may induce endothelial dysfunction and downstream luminal contraction (Zhou et al., 2023; Lim et al., 2020). The high–kinetic energy jet persistently impacting the distal vessel wall poses a risk of endothelial injury (Xing et al., 2025; Zhu et al., 2024). Distal to the stenosis, pressure and flow reduction led to flow deceleration, dispersion, and vortical structures, reflecting disturbed hemodynamics.

The trans-stenotic pressure gradient, a key quantitative indicator of PVS severity, was markedly elevated preoperatively, indicating severe hemodynamic compromise. Consistent with previous CFD studies (Riedel et al., 2024; Xiong et al., 2024), this gradient reflects substantial energy loss across the stenosis, with dissipation of kinetic energy as heat and mechanical stress. Such hemodynamic inefficiency may reduce hepatic parenchymal perfusion and impair graft regeneration and function (Kawaguchi et al., 2013). Accordingly, timely endovascular intervention was warranted.

At 1 week post-surgery, CFD simulations demonstrated restoration of luminal area attenuated jet flow and reduced the trans-stenotic pressure gradient. The post-surgery normalization of WSS suggests re-establishment of a physiologically favorable mechanical environment, potentially supporting vascular remodeling and long-term patency. These findings indicate that balloon angioplasty successfully alleviated the vascular stenosis, resulting in restored hemodynamic homeostasis and improved PV perfusion.

Beyond alleviating focal stenosis, balloon angioplasty significantly altered flow distribution within the intrahepatic portal venous branches, characterized by increased flow in the right posterior branch and a corresponding decrease in the right anterior branch. This flow redistribution may result from multiple factors, including PV morphology, hemodynamic effects, intrahepatic vascular remodeling, and changes in hepatic parenchymal resistance. In the early postoperative period, it was primarily driven by mechanical relief of the stenosis and hemodynamic changes at the bifurcation; over time, however, intrahepatic vascular remodeling mediated by graft regeneration and dynamic changes in hepatic parenchymal resistance also affect flow redistribution. These findings indicate that PVS represents a system-level hemodynamic disorder rather than a purely focal lesion, as localized resistance changes can propagate downstream and remodel intrahepatic perfusion patterns. Study has shown that the blood flow of the PV branches is positively correlated with the corresponding liver parenchymal volume (Xie et al., 2024), and alterations in perfusion may influence graft regeneration. Thus, dynamic monitoring of portal branch flow may provide a useful indicator of regional hepatic regeneration.

At 6 months post-surgery, the trans-stenotic pressure gradient further decreased, and the overall WSS distribution became more uniform, indicating sustained therapeutic efficacy. These hemodynamic improvements paralleled the clinical symptomatic relief, demonstrating the sensitivity of CFD in reflecting the physiological outcomes of surgery.

The hemodynamic status at 1 week post-surgery reflects the immediate geometric restoration of the stenotic segment, as evidenced by marked reductions in velocity ratios and trans-stenotic pressure gradients. Follow-up at 6 months revealed persistent evolution in flow patterns, wall shear stress, and flow distribution across the portal venous system. These findings suggest a time-dependent adaptive interplay between vascular geometry and blood flow, underscoring the necessity of longitudinal hemodynamic assessment when evaluating the efficacy of interventional treatment for PVS after LDLT.

The values of this study are as follows: First, when conventional imaging (CTA or Ultrasound) does not fully explain a patient’s clinical symptoms or graft dysfunction, our CFD framework can provide complementary insights by quantifying metrics such as trans-stenotic pressure gradients, WSS, and intrahepatic branch flow. Second, balloon angioplasty alters intrahepatic branch flow distribution. Given that portal branch flow correlates with liver parenchymal volume (Xie et al., 2024), tracking these changes offers a noninvasive window into regional graft perfusion and regeneration dynamics. Importantly, this framework enables the longitudinal monitoring of postoperative hemodynamic trends in PVS patients and may assist clinicians in identifying potential risks of disease progression prior to the manifestation of clinical symptoms. Together, these contributions lay the groundwork for establishing a standardized PVS assessment framework incorporating multi-patient datasets.

Several limitations of this study should be acknowledged. First, as a proof-of-concept study, this investigation was limited to a single patient and warrants future large-scale, multicenter studies to validate its clinical generalizability. Second, the accuracy of CFD analysis depends on model fidelity. Several simplifying assumptions—such as assuming rigid vessel walls and neglecting pulsatile flow—may affect the precision of the simulations. While absolute values may vary, the relative magnitude of hemodynamic improvement remains a reliable indicator of procedural success. Future studies could enhance physiological realism by incorporating fluid–structure interaction pulsatile flow models. Third, the complexity of CFD modeling and computational requirements currently limits its routine clinical application. The development of user-friendly, clinically oriented software platforms will be essential to promote wider adoption and integration into postoperative surveillance workflows. Despite these limitations, the present modeling framework captures the dominant hemodynamic features associated with PVS and its endovascular treatment.

## 5 Conclusion

This patient-specific proof-of-concept CFD study quantitatively assessed the longitudinal hemodynamic recovery following balloon angioplasty for PVS after LDLT. Over a 6-month follow-up, the surgery successfully normalized key hemodynamic metrics: the PVV ratio decreased from 3.21 to approximately 1.01, the trans-stenotic pressure gradient dropped from 197.13 Pa to 4.67 Pa, and peak WSS reduced from 30.21 Pa to 0.77 Pa. Despite its limitations, this framework yields critical functional insights unattainable by conventional imaging. As an effective quantitative tool for tracking serial hemodynamic trends, it may facilitates evaluation of disease changes and therapeutic response. Validating these CFD-derived parameters against clinical outcomes in large-scale, multi-patient studies is essential to establish their predictive value. Such extensive validation is a prerequisite for considering this framework’s clinical translation in post-LDLT PVS management.

## Data Availability

The original contributions presented in the study are included in the article/supplementary material, further inquiries can be directed to the corresponding author.
